# Spatial and temporal distribution characteristics and geographic contexts of civilized villages in China

**DOI:** 10.1371/journal.pone.0305591

**Published:** 2024-06-24

**Authors:** Qiyan Ding, Zhen Yang, Zhouyu Huang

**Affiliations:** 1 College of Urban and Environmental Sciences, Central China Normal University, Wuhan, Hubei, China; 2 Key Laboratory for Geographical Process Analysis & Simulation of Hubei Province, Central China Normal University, Wuhan, Hubei, China; Peking University, CHINA

## Abstract

Rural civilization is the soul of rural revitalization strategies. As a booster of rural civilization, the civilized village is an indispensable force to promote the modernization of rural areas, in the meanwhile, the study of its spatial distribution has important theoretical significance and practical value in deepening the theory of rural geography, promoting the development of rural civilization, and helping rural revitalization. Considering civilized villages as the study topic, the spatial and temporal distribution characteristics and geographic context were discussed using spatial analysis methods. The number of civilized villages in the country has shown a clear upward trend since 2005. The spatial distribution pattern of civilized villages in China shows unbalanced patterns with a higher concentration in the east and south. Civilized villages in China present significant patterns of clustering with an increasing degree of spatial clustering, showing a multi-core spatial distribution pattern. Civilized villages in China demonstrate clear spatiotemporal clustering characteristics. A spatiotemporal hot spot cluster formed in the northwestern region from 2005 to 2011, and a spatiotemporal cold spot cluster formed in the southeastern region from 2005 to 2008. The spatial distribution of civilized villages in China has obvious geographical differentiation laws, and factors such as topography, climate, economy, transportation, and policy significantly affect their spatial distribution.

## 1. Introduction

If the countryside grows, the country prospers; if it declines, the country declines. To build a moderately prosperous society and a strong modern socialist country, the most arduous and onerous task lies in the countryside; the broadest and deepest foundation lies in the countryside, and the greatest potential and staying power also lies in the countryside [1]. However, some villages currently face problems such as outdated customs, moral decline, feudal superstitions, and agriculture-related crimes, which seriously restrict the growth of rural districts, hence the construction of rural civilization is urgent [2]. The 19th Party Congress proposed the rural revitalization strategy, taking “rural civilization” as one of the general requirements of the rural revitalization strategy. The Strategic Plan for Rural Revitalization (2018–2022) issued by the Central Committee of the Communist Party of China and the State Council in 2018 pointed out that “rural revitalization is guaranteed by rural civilization.” “It is necessary to insist on material and spiritual civilization together; cultivate a civilized countryside, a good family culture, and simple folk customs; and constantly improve the degree of civilization in rural society.” In the report of the 20th Party Congress, the Central Committee of the CPC once again proposed the goals of “raising the level of civilization of the whole society” and “promoting the fusion development of spiritual civilization construction among rural and urban areas.” It can be seen that rural civilization is an indispensable part of the rural revitalization strategy, and is the soul of rural revitalization. In the construction of rural civilization, civilized villages are important carriers, and the broad masses of peasants constantly pursue a high-quality spiritual life from the establishment of civilized villages, which fosters the comprehensive development of rural revitalization. Therefore, a systematic study of the spatial distribution characteristics of civilized villages and their geographic context from a global perspective is of great practical significance for governments in guiding the construction of rural civilization, selecting and cultivating models of rural civilization, and promoting rural revitalization.

With the promotion of rural revitalization strategies and central governmental attention to rural work, research on rural civilization has become an important topic. Currently, academic research mainly focuses on the significance, content, problems, paths, and other aspects of rural civilization construction [3–9]. For example, in terms of significance, it has been pointed out that rural civilization is the soul, guarantee, and core content of rural revitalization, which plays a major role in raising the lives of farmers and facilitating fusion development among urban and rural areas [3], with rich results and different views that provide useful references for future related research. In terms of content, it mainly includes the leadership of party building, curbing bad habits, innovation of guidance, construction of positions, driving force of activities, leadership of culture, and a typical model [4]. In terms of challenges, it is believed that there are some problems in the process of rural civilization construction, such as the low overall quality of farmers, incomplete understanding of grassroots cadres, lack of material support, and an imbalance in rural cultural development [5, 6]. In terms of paths, it is proposed that the construction of rural civilization should be driven by the implementation of rural public cultural projects, carrying out activities of changing customs and habits, inheriting and carrying forward advanced rural culture, and optimizing the structure of financial investment [7–9]. However, from existing results, the study of rural civilization is primarily based on the perspectives of sociology, management, and political science, and few studies have been conducted from geographical perspective.

Regarding the study of rural civilization, although geography does not have a clear research theme, some scholars have explored the degree of regional rural civilization construction in the field of rural revitalization and achieved some results [10–13], that have not been studied in depth because rural civilization is only one aspect of rural revitalization. A village is the smallest settlement unit, has a geographical association with natural, social, and economic characteristics, and is an essential carrier of the rural revitalization strategy [14]. In the process of promoting rural civilization in the new era, many outstanding examples of national village-level rural civilization construction have emerged. As typical villages for the construction of rural civilizations, civilized villages are an entry point for geographical research on rural civilizations. Exploring the distribution characteristics of civilized villages in China and their geographic context from spatial and temporal perspectives and revealing the geographic differentiation law of their distribution is conducive to enriching geographic research and expanding current research perspectives on rural civilization. At present, there are few studies on the spatial distribution of civilized villages, but some scholars have conducted extensive research on typical villages related to rural revitalization selected by the country, such as professional villages [15–18], model villages for rural revitalization [19, 20], beautiful villages [21, 22], rural tourism villages [23, 24], rural governance demonstration villages [25], traditional villages [26–28], ethnic minority villages [29], and forest villages [30], and have studied the spatial distribution characteristics and influencing factors of various typical villages from different perspectives. This has created a foundation for exploring the spatial distribution of various typical villages for rural revitalization and provided a reference for the research on the spatial distribution of civilized villages in China.

A large number of studies on the spatial distribution of typical villages in geography can be found in existing literature. Nonetheless, relevant research on civilized villages in China is insufficient. Research on civilized villages can enrich knowledge on rural civilization and provide a basis for its construction. In addition, this study analyzes the distribution characteristics of civilized villages based on integrated temporal, spatial, and spatiotemporal perspectives, which can determine the situation of the construction of rural civilization accurately and comprehensively. To achieve rural revitalization in China, it is necessary to guarantee countryside civilization. As models of rural civilization, civilized villages play an important role in guiding rural revitalization. Based on this, this study takes civilized villages assessed by the China Civilization Office from 2005 to 2020 as the research object and comprehensively applies the nearest neighbor index, kernel density, and spatiotemporal scanning statistics to investigate the spatial and temporal characteristics of their distribution. It also explores the geographic context features of civilized villages in China in terms of nature and humanities to offer a basis for optimization of the spatial structure of such villages, provides references for the monitoring and identification of civilized villages in China, and enriches related research content of rural geography.

## 2. Materials and methods

### 2.1. Data sources

The civilized villages in China is an honorary title granted by the Central Spiritual Civilization Construction Steering Committee, and the villages awarded with this honorary title are the administrative villages with the coordinated development of “two civilizations,” outstanding achievements in the construction of spiritual civilization, and a typical demonstration in the country [31]. The first session on the selection and commendation of civilized villages was launched in 2005. Since then, selection activities have been carried out in an orderly manner every three years, and up to now, six sessions of selection have been conducted. After excluding some villages in the duplicate lists, 4824 civilized villages remained. The data of civilized villages came from the list published by the China Civilization Network (http://www.wenming.cn/). Referring to existing research [14], each civilized village was abstracted as a point, and the location information and coordinates were found one by one through the Baidu coordinate pickup system, and then the acquired geographic coordinates were imported into ArcGIS10.2 software to carry out the alignment and construct the spatiotemporal distribution and attribute database of civilized villages in China. The geographic contextual factors of civilized villages were analyzed from both natural and humanistic social aspects. The spatial distribution raster data for elevation, slope, annual average temperature, precipitation, and GDP were obtained from the Resource and Environment Science and Data Center of the Chinese Academy of Sciences. Owing to data availability, the 2019 grid data was selected as the GDP data. Road traffic network data was obtained from the AMap road traffic network for the year 2020.

### 2.2. Research methods

#### 2.2.1. Nearest neighbor index

The nearest neighbor index calculates the state of proximity of point elements in geographical space by measuring the average distance between the target point and the nearest neighbor point [32]. It is used to analyze the approaching state of civilized villages in China, the formula is:

$$L=L_{i}/L_{e}=\frac{1}{m}\sum_{i=1}^{n}d_{i}\left(s_{i}\right)\times \frac{1}{2\sqrt{m/Z}}$$ (1)

*L* notes the nearest neighbor index; *L*_*i*_ denotes the actual average observed distance; *L*_*e*_ is the expected nearest neighbor distance; *d*_*i*_*(s*_*i*_*)* denotes the distance from a point to its nearest neighbor point; *m* notes the number of civilized villages in the country; *Z* denotes the total territory of the research region. When *L*>1, the spatial distribution of civilized villages in China is discrete; when *L*<1, it represents clustering; when *L* = 1, it shows that is random.

#### 2.2.2. Standard deviation ellipse

The standard deviation ellipse can measure the direction, morphology and average center of the spatial distribution of point [33]. The working principle is to determine the axis of an ellipse by calculating standard deviation of the x-coordinate and y-coordinate of a set of elements from the average center. In order to analyze the distribution direction and morphology of civilized village in China, the standard deviation ellipse method is introduced to disclose the change in distribution range and direction. The calculation formula is:

$$\left\{\begin{array}{l} {\tilde{x}}_{i}=\left(x_{i}-\bar{x}\right)\\ {\tilde{y}}_{i}=\left(y_{i}-\bar{y}\right) \end{array}\right.$$ (2)

$$G=\frac{1}{m}\left(\begin{array}{ll} \sum_{i=1}^{m}{{\tilde{x}}_{i}}^{2} & \sum_{i=1}^{m}{\tilde{x}}_{i}{\tilde{y}}_{i}\\ \sum_{i=1}^{m}{\tilde{x}}_{i}{\tilde{y}}_{i} & \sum_{i=1}^{m}{{\tilde{y}}_{i}}^{2} \end{array}\right)$$ (3)

(*x*_*i*_, *y*_*i*_) represents the coordinates of civilized village *i* (*i* = 1,2,3,…,*m*); ($\bar{x}$,$\bar{y}$)denotes the average center;${\tilde{x}}_{i}$, ${\tilde{y}}_{i}$ respectively denote the coordinate deviation from each village point to the average center; *G* is the flatness of the standard deviation ellipse; *m* is the total number of points.

#### 2.2.3. Kernel density

Kernel density is a non-parametric estimation model devoted to describing spatial density characteristics and distribution trends [34], which can clearly reflect the spatial clustering or dispersion features of civilized village in China, and its formula is:

$$f\left(x\right)=\frac{1}{mw}\sum_{i=1}^{m}k\left(\frac{x-x_{i}}{w}\right)$$ (4)

$\left(\frac{x-x_{i}}{w}\right)$ represents the functional form; *x* is the location of civilized village point; *x-x*_*i*_ expresses the length from the estimated point to the sample point; *w* is the bandwidth (*w*>0); and *m* means the number of points in the bandwidth range.

#### 2.2.4. Spatiotemporal rearrangement scanning statistical method

The specific detection process of the spatiotemporal rearrangement scanning statistical method uses a cylindrical scanning window, the center of its bottom surface corresponds to a point in the detected geographic region, the height matches the corresponding time interval, the semi-diameter of the bottom surface gradually increases, so that the scanned spatial region constantly expands until the set maximum radius, at the same time, the cylinder’s height is also gradually increased, add a specified time unit each time until the preset upper limit is reached [35]. The change process of the scanning window is repeated throughout the study area, which will result in a large number of scanning windows. The generalized likelihood ratio is used to judge whether the number of cases within each scanning window is anomalous or not, and its value reflects the possibility of the window being clustered, and the window with the largest function value is most likely to be clustered. Then, the Monte Carlo hypothesis testing method is used to analyze the confidence of non-randomness of the obtained candidate agglomeration areas, and finally obtain a reasonable agglomeration area [36]. The step of calculating the spatiotemporal clustering of civilized villages in China using the spatiotemporal rearrangement scanning statistical method is as follows [37]:

Let *z* represent a certain region and *d* represent a certain period. The number of civilized villages in a certain region *z* and a certain period *d* is *C*_*z*,*d*_, then the number of all civilized villages *C* in all regions and time ranges is:

$$C=\sum_{z}\sum_{d}C_{z,d}$$ (5)

The expected values for each unit area and unit time *μ*_*z*,*d*_ are:

$$\mu_{z,d}=\frac{1}{C}(\sum_{z}C_{z,d})(\sum_{d}C_{z,d})$$ (6)

$\sum_{z}C_{z,d}$ is the quantity of civilized villages in the entire study district during *d* period; $\sum_{d}C_{z,d}$ is the quantity of civilized villages in the entire time span of sector *z*.

Then the expected number of civilized villages within the cylinder scanning window *A* is:

$$\mu_{A}=\sum_{(z,d)\in A}\mu_{z,d}$$ (7)

Let *C*_*A*_ be the actual number of civilized villages in the cylindrical window *A*, *C*_*A*_ obeying a hypergeometric distribution with mean *μ*_*A*_, whose probability function is:

$$P(C_{A})=\left(\begin{array}{l} \sum_{z\in A}C_{z,d}\\ C_{A} \end{array}\right)\left(\begin{array}{l} C-\sum_{z\in A}C_{z,d}\\ \sum_{d\in A}C_{z,d}-C_{A} \end{array}\right)/\left(\begin{array}{l} C\\ \sum_{d\in A}C_{z,d} \end{array}\right)$$ (8)

When $\sum_{z\in A}C_{z,d}$ and $\sum_{d\in A}C_{z,d}$ are very small relative to *C*, the approximation of *C*_*A*_ obeys Poisson distribution with mean value *μ*_*A*_. Based on this, the Poisson generalized likelihood function can be used to determine whether civilized villages in the cylinder window *A* are clustered or not, and the expression of the generalized likelihood ratio (*GLR*) is:

$$GLR={\left\{\frac{C_{A}}{\mu_{A}}\right\}}^{C_{A}}{\left\{\frac{C-C_{A}}{C-\mu_{A}}\right\}}^{C-C_{A}}$$ (9)

## 3. Results

### 3.1. Evolutionary characteristics of the spatial and temporal pattern of civilized villages in China

#### 3.1.1. Spatial and temporal distribution pattern

**Fig 1:**
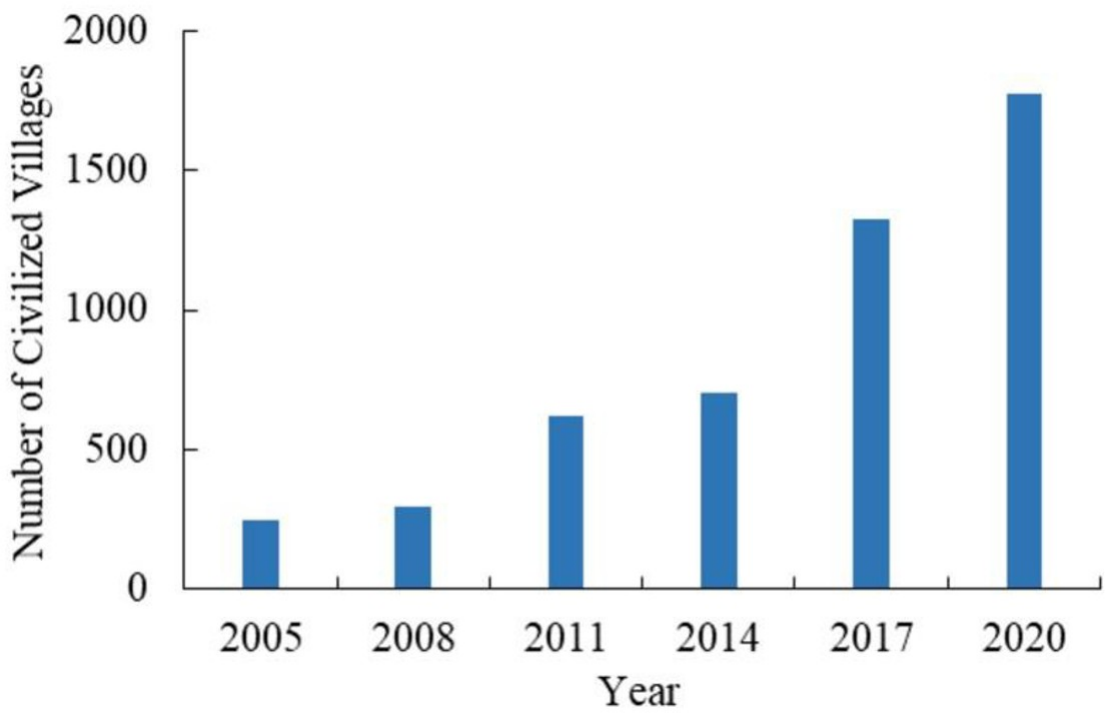
Change of the number of civilized villages in China.

The number of civilized villages in China from 2005 to 2020 was counted and analyzed (Fig 1). In terms of quantity, civilized villages presented an evident upward trend. In 2005, 245 were selected, while in 2008, 2011, 2014, and 2017, 295, 619, 701, and 1323 were selected respectively. In 2020, 1772 were selected, and this number continues to increase. In terms of growth rate, civilized villages present a trend of rapid growth. In 2008, the number of civilized villages increased by 50 compared to 2005, while in 2017, the number of selected villages increased by 622 compared to 2014. In 2020, it increased by 449 compared to 2017, revealing that the construction of rural civilization is in a rapid development stage.

**Fig 2:**
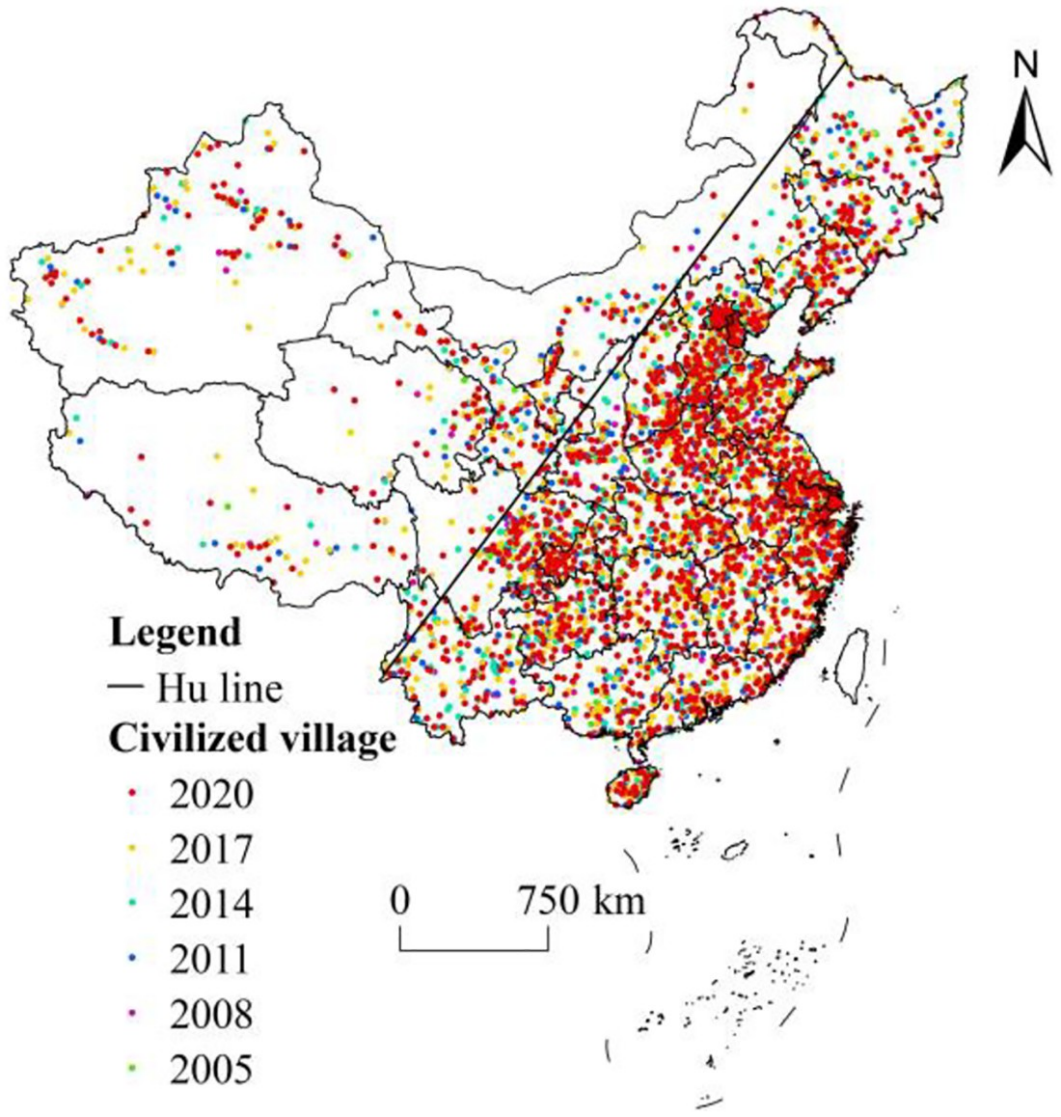
Spatial distribution of civilized villages in China. Note: The base map came from Natural Earth (http://www.naturalearthdata.com/).

The “Hu Huanyong Line” and the “Qinling-Huaihe River” are two important geographic boundaries in China, with obvious differences in natural conditions and population density on both sides of the boundaries, and they are dividing lines between east-west and north-south of China. To characterize their spatial distribution, civilized villages were spatially superimposed and statistically partitioned with two basic geographic boundaries. The analyses demonstrate that the civilized villages in China present a spatial pattern of more in the east and less in the west, more in the south and less in the north (Fig 2). Bounded by the “Hu Huanyong Line,” the number of civilized villages to the east of the line were 4346, accounting for 90.09%, with a high spatial distribution density. West of the line, civilized villages were 478, accounting for only 9.91%, with a more dispersed distribution. Taking “Qinling-Huaihe River” as the boundary, there were 2561 civilized villages south of the boundary, accounting for 53.09%, and 2263 civilized villages north of the boundary, accounting for 46.91%, presenting a spatial distribution of more in the south and less in the north. Overall, the spatial spread of civilized villages across the country is significantly different from east to west than from north to south.

On the provincial scale, civilized villages have spread across provinces in China (except Hong Kong, Macao, and Taiwan), but there is a large gap between the provinces. To date, civilized villages in Hebei Province have been obtained the most times, with a total of 299, while Tibet, with only 50, has the smallest, with a difference of 249 between the two provinces. At the city scale, Shijiazhuang, Changzhi, Jincheng, Baoding, Xingtai, Tangshan, Handan, and Huanggang have become cities where civilized villages are clustered and spread out to the periphery, with these as the centers. At the county scale, the spatial agglomeration of civilized villages is ordinary, with more civilized villages in Wuqing, Pudong, Jinghai, Jizhou, and Baodi.

#### 3.1.2. Spatial and temporal differentiation characteristics

To dissect the level of mutual proximity of civilized villages in China and judge their spatial distribution types, the nearest neighbor indices were calculated for each session of civilized villages using ArcGIS10.2 respectively, the findings are shown in Table 1. From the table, the average observed distances of each session of civilized villages are smaller than the expected average distances, and the values of the nearest neighbor index are less than 1, which indicate that this type of village shows a significant spatial clustering pattern. From the change in value, the result reveals a decreasing trend in general, from 0.6638 to 0.6145, indicating that the degree of agglomeration of civilized villages has been enhanced over time. The average observation distance shrinks substantially, from 81978m to 30062m, which is far less than the expected average distance. From the change in *Z*-score, all *Z*-scores are less than -2.58, and decrease year by year, from -10.07 to -31.04, with *P*-values less than 0.01, significant at the 1% level.

**Table 1:** Nearest neighbor index of civilized villages in China.

Year	Average Observed Distance (m)	Expected Average Distance (m)	Nearest Neighbor Index	*Z* Score	*P* Value	Type of Spatial Distribution
2005	81978	123497	0.6638	-10.07	<0.01	agglomeration
2008	76821	116213	0.6610	-11.14	<0.01	agglomeration
2011	51425	81374	0.6320	-17.52	<0.01	agglomeration
2014	48217	77111	0.6253	-18.98	<0.01	agglomeration
2017	33962	57481	0.5909	-28.47	<0.01	agglomeration
2020	30062	48920	0.6145	-31.04	<0.01	agglomeration

**Fig 3:**
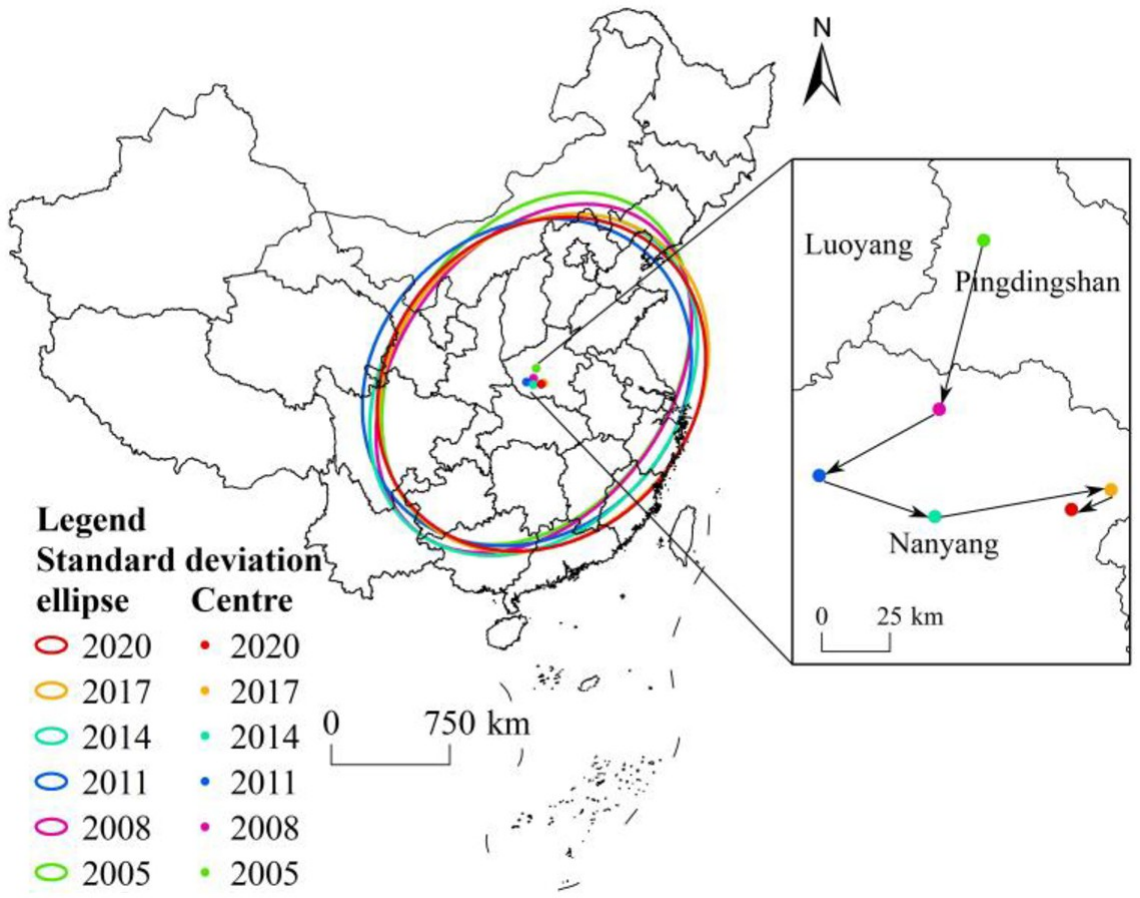
Gravity shift trajectory and standard deviation ellipse of civilized villages in China. Note: The base map came from Natural Earth (http://www.naturalearthdata.com/).

To determine variations in the spatial pattern of civilized villages in China, the center of gravity of the distribution was taken as the center and the standard deviation ellipse method was used to estimate the clustering range, directional alteration, and center of gravity migration characteristics of each civilized village session. The center-of-gravity migration trajectory and ellipse were plotted in Fig 3. The findings indicate that civilized villages in China from 2005 to 2020 were relatively concentrated, but there were also some differences.

In terms of the degree of agglomeration, the overall area of ellipsoids did not change significantly since 2005, and the spatial agglomeration distribution was relatively stable (Table 2). In terms of distribution direction, the ellipses are mainly located in the eastern section of China, with a “northeast-southwest” direction, roughly consistent with the direction of the “Hu Huanyong Line.” In terms of the center of gravity and migration directions, the center of gravity of civilized villages are mainly located in Pingdingshan City and Nanyang City in Henan Province, moving from (112.74°E, 34.02°N) to the southwest (112.01°E, 33.29°N), then to the southeast (113.17°E, 33.17°N), and to the southwest (113.01°E, 33.12°N), and the overall directions of development are towards the east and south. On the whole, the standard deviation ellipses of civilized villages do not change much, the center of gravities are always in Henan, and they are distributed in the direction of “Northeast-Southwest,” which further verifies the consistency between its overall distribution and the “Hu Huanyong Line.” The distribution of civilized villages was denser in the inner part of the ellipse and sparser in the outer part.

**Table 2:** Standard deviation ellipse calculation results of civilized villages in China.

Year	Perimeter (km)	Area (10000km^2^)	Longitude of Center of Gravity (°E)	Latitude of Center of Gravity (°N)	X-Axis Standard Deviation (km)	Y-Axis Standard Deviation (km)	Rotation Angle (°)
2005	6529.11	326.18	112.74	34.02	866.23	1198.68	33.03
2008	6542.63	324.93	112.51	33.48	850.89	1215.61	36.69
2011	6495.35	332.70	112.01	33.29	952.11	1112.34	46.19
2014	6611.67	338.77	112.46	33.13	908.89	1186.50	40.44
2017	6602.64	341.84	113.17	33.17	944.61	1151.97	41.28
2020	6558.47	338.95	113.01	33.12	958.08	1126.19	42.25

**Fig 4:**
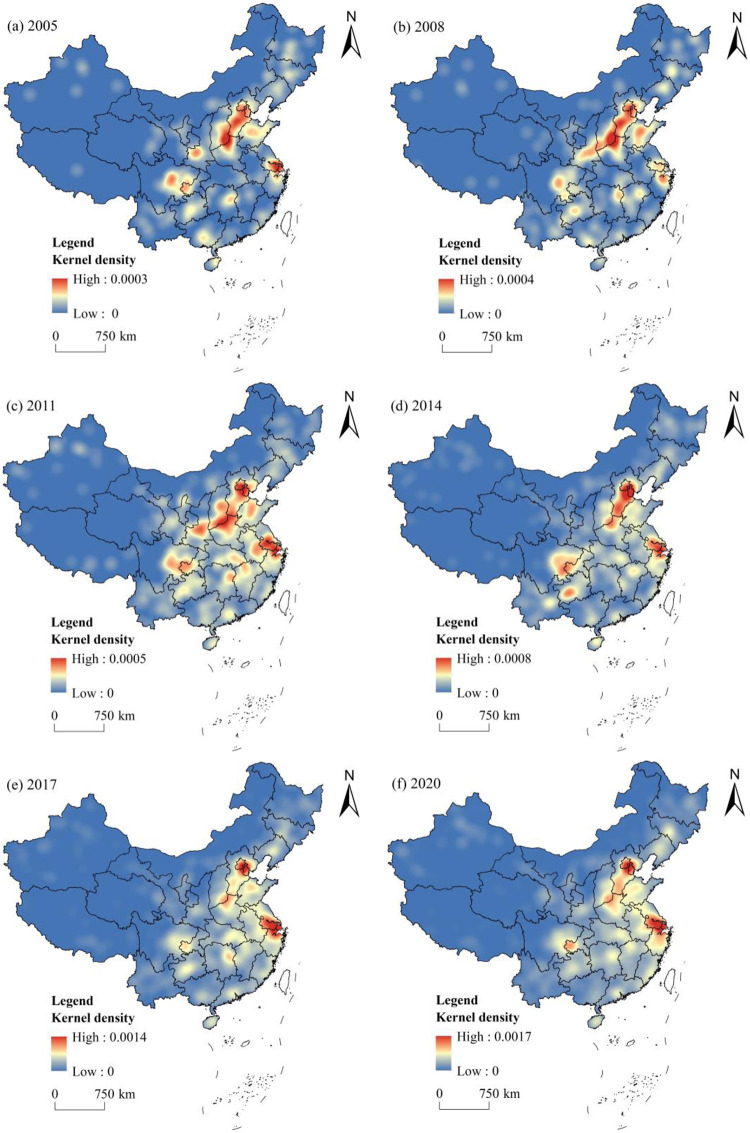
Kernel density of civilized villages in China. Note: The base map came from Natural Earth (http://www.naturalearthdata.com/).

To further reveal the evolutionary process and spatial agglomeration distribution characteristics of civilized villages in China, kernel densities in 2005, 2008, 2011, 2014, 2017, and 2020 were determined, and the findings are shown in Fig 4. The kernel density value of civilized villages from 2005 to 2020 demonstrated an increasing trend, with the highest value increasing from 0.0003 to 0.0017, indicating that its distribution is becoming increasingly dense. At the same time, it is also displayed that the spatial agglomeration distribution of civilized villages developed unevenly in general, presenting a spatial distribution pattern of multiple cores, mainly forming high-density areas dominated by Beijing-Tianjin-Hebei, the Yangtze River Delta, Sichuan-Chongqing, and the junction areas of Shanxi and Henan. Comparing the estimated values of kernel density, the value of kernel density in the eastern area was higher than that in the western area, indicating that the agglomeration intensity of civilized villages was higher in the eastern region and lower in the western region. Since 2005, the high-density areas of kernel density have expanded significantly outward and gradually spread to the surface from the initial point-like distribution.

#### 3.1.3. Spatiotemporal agglomeration characteristics

Currently, widely used spatial autocorrelation analysis methods do not incorporate time factors and cannot judge the trend of geospatial agglomeration area over time, which have certain limitations. However, spatiotemporal scanning statistical analysis simultaneously considers both time and space, which can reveal the law of the factor agglomeration area changing with time in geospatial space. Compared to spatial autocorrelation analysis, it can accurately locate the spatiotemporal agglomeration area of geographic elements [38]. The spatiotemporal scanning statistics clustering method has been primarily applied in the field of epidemiology [39–41]. With gradual development and improvement of the scanning statistics model, this method has been widely applied in geography and some analysis [42, 43]. SaTScan software is currently a representative spatiotemporal scanning statistics software, which can conduct geographic monitoring of research objects, explore spatial or spatiotemporal agglomerations, and analyze whether there is statistical significance. It can also test whether a study object is random in terms of its time, space, and spatiotemporal distribution [44]. Therefore, SaTScanV9.5 software was used to calculate the spatiotemporal rearrangement scanning of civilized villages in China and detect statistically significant spatiotemporal agglomeration.

Spatiotemporal rearrangement scanning statistics methods are divided into two types: retrospective and prospective. Because the goal of this study is to discover spatiotemporal agglomeration features of the distribution of civilized villages, retrospective spatiotemporal rearrangement scanning statistics were chosen as the agglomeration detection method. As the basic unit of national governance, a county is at the core of the national governance system. Compared to other scales, it has unique advantages and can better discover the spatiotemporal agglomeration characteristics of civilized villages. Therefore, we applied spatiotemporal rearrangement scanning statistics on a county basis. Spatiotemporal rearrangement scanning statistical analysis was conducted on civilized villages in China from 2005 to 2020. The input data were the number of civilized villages in each county, temporal data, and geographic coordinates; 50% of the total study period was taken as the temporal scale, and 3-year was taken as the unit. The statistical test was conducted through a Monte Carlo simulation, and the statistical results of the spatiotemporal scanning agglomeration were obtained (Table 3). The results indicated that the hot and cold spot agglomeration areas both had a region that passed the 95% confidence test.

**Table 3:** Statistics of spatiotemporal clustering of civilized villages in China.

Type	Main Gathering Area	Gathering Time (year)	Central Point (Lng, Lat)	Gathering Radius (km)	Observed Value (pcs)	Expected Value (pcs)	*P* Value
Hot spot	Anse, Zhidan, Zichang, Ganquan, Mizhi, etc	2005–2011	36.92N,109.16E	458.08	185	125	0.001
Cold spot	Wuning, Jing’an, Tongshan, Ruichang, Yongxiu, etc	2005–2008	29.26N,115.01E	608.53	92	155	0.002

**Fig 5:**
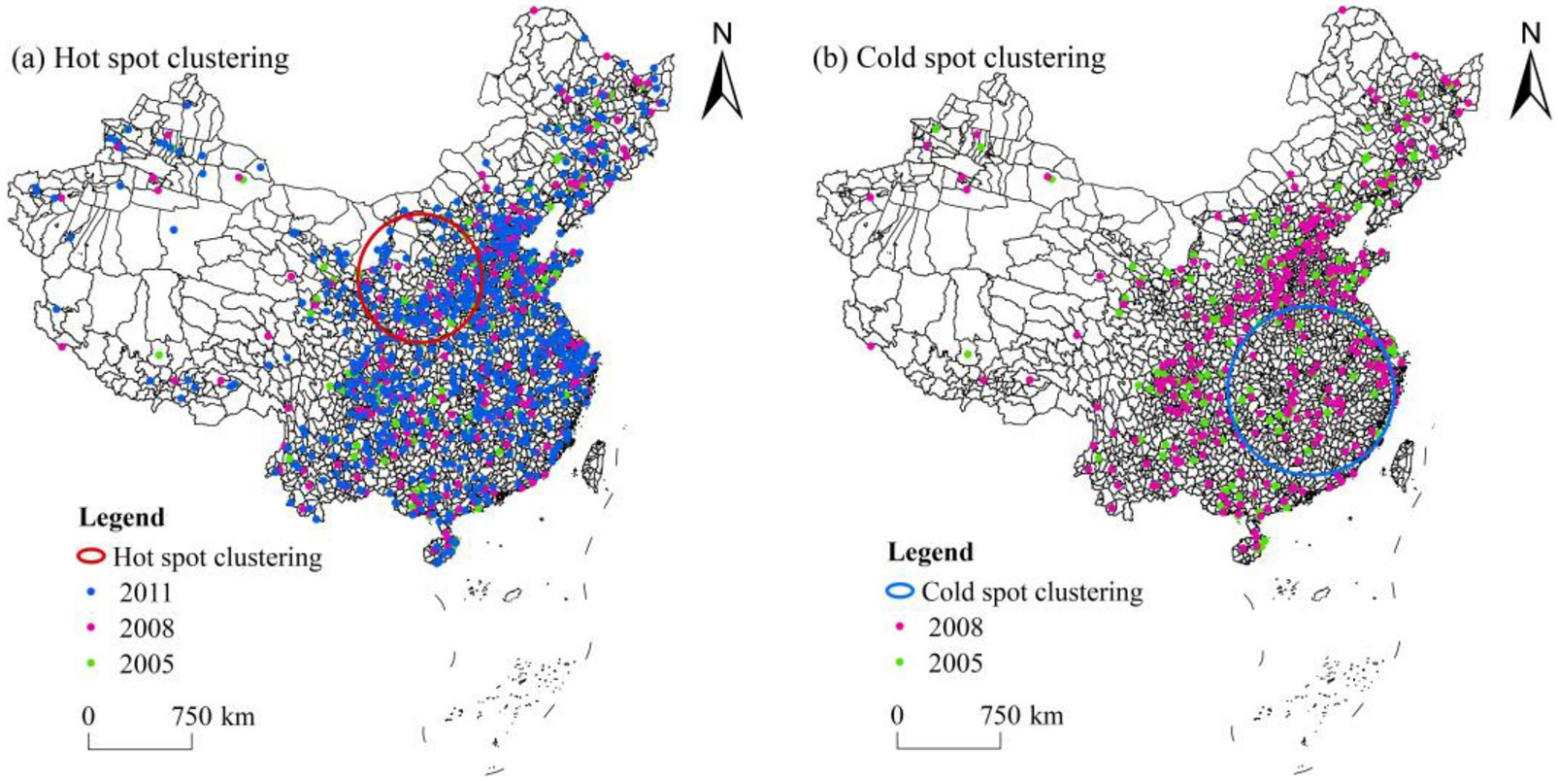
Hot and cold spot distribution of spatiotemporal scan of civilized villages in China. Note: The base map came from Natural Earth (http://www.naturalearthdata.com/).

Using ArcGIS10.2 software for visual expression, the results of the spatiotemporal scanning agglomeration of the civilized villages in China are plotted (Fig 5). Civilized villages are not randomly distributed but present obvious spatiotemporal clustering characteristics. Further analysis of the distribution characteristics of the spatiotemporal scanning results revealed that a spatiotemporal hot spot agglomeration area formed in northwest China from 2005 to 2011 (Fig 5a), mainly located in the Anse District, Zhidan County, Zichang City, Ganquan County, Mizhi County, and other areas, with a coverage radius of 458.08 km. There were 185 civilized villages in the area, more than expected. Although there were not many civilized villages in the hot spot agglomeration area, the growth rate of civilized villages in this area was higher than that of the surrounding areas from 2005 to 2011; therefore, this area became a hot spot for spatiotemporal agglomeration. A spatiotemporal cold spot agglomeration area was formed in the southeastern of China from 2005 to 2008 (Fig 5b), mainly located in Wuning County, Jing’an County, Tongshan County, Ruichang City, Yongxiu County and other regions, with a covering radius of 608.53 km. In 2005, the number of civilized villages in this region was 38, compared to 54 in 2008, an increase of 16, far less than expected. The growth rate was slower than that in surrounding areas, making it a cold spot for spatiotemporal agglomeration.

### 3.2. Analysis of the geographic context of civilized villages in China

According to the above research, there was a fuller understanding of the distribution pattern of civilized villages, and it was found that their spatial distribution pattern is not random, but closely related to the geographic environment. Existing research results also imply that the spatial distribution of various typical villages is affected by multiple factors, such as natural factors, social economy, transport location, distinctive culture, and national policies [15, 19, 21, 24, 27]. Based on existing research, considering the accessibility, correlation, and scientificity of indicator data, the geographic context of the spatial distribution of civilized villages was analyzed from the terrain, climatic conditions, economic conditions, distance to counties (towns) centers, and distance to national (provincial or county) roads.

**Fig 6:**
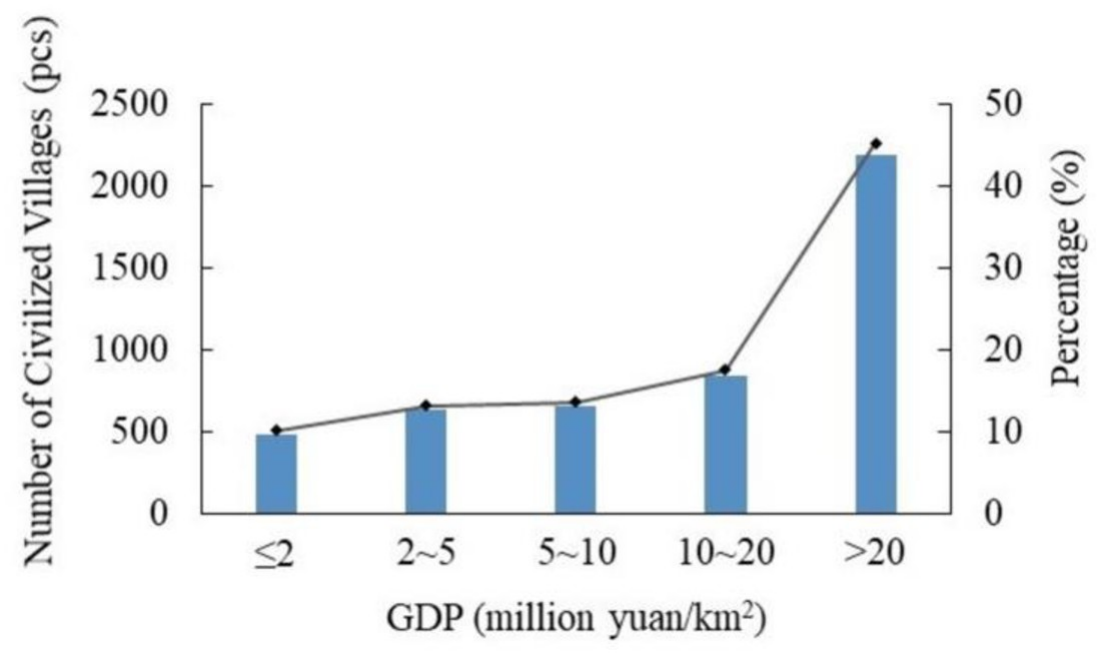
Classification statistics of GDP of civilized villages in China.

**Fig 7:**
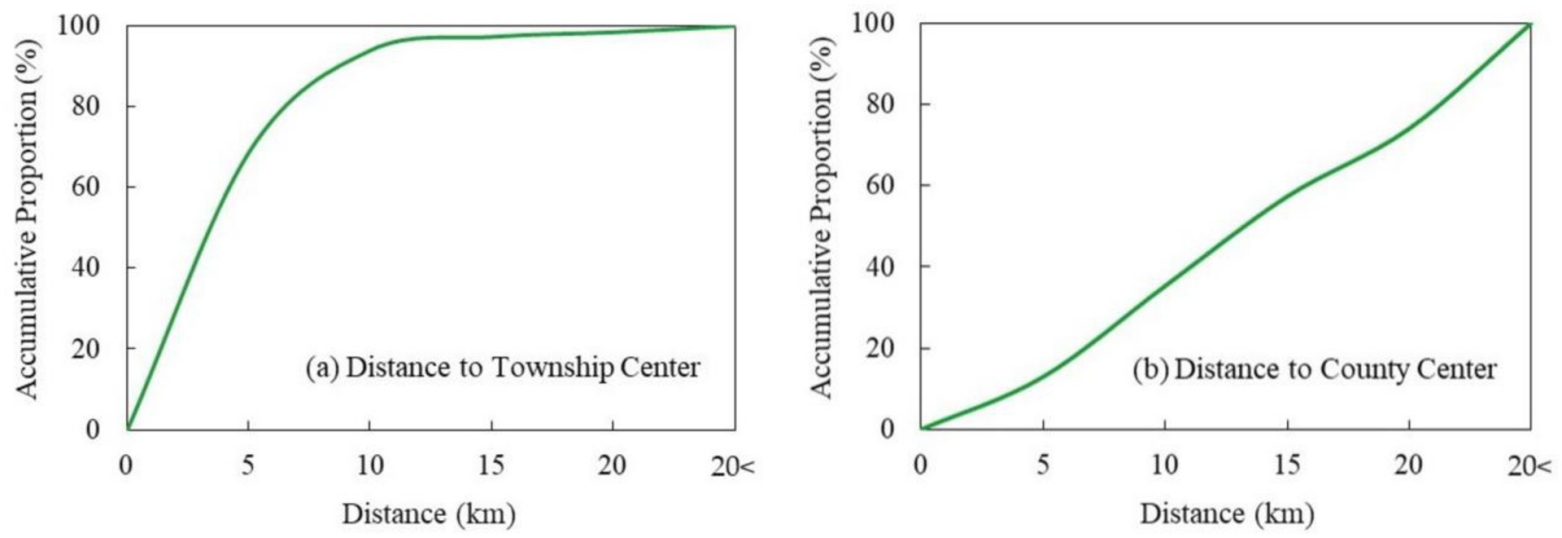
The accumulative proportion of civilized villages at different centers in China.

(1) Topography and geomorphology. Using the spatial coupling of civilized villages with elevation and slope in China, we found that they were primarily distributed in places with low elevation and small slopes (Table 4). In terms of elevation distribution, 96.25% of the civilized villages were distributed in areas below 2000m above sea level. The number of civilized villages in the plain and hilly areas below 500m was the highest, accounting for 70.42%; in the areas of 500~1000m, accounting for 12.40%; and in the areas of 1000~1500m and 1500~2000m, the number was relatively small, accounting for 9.81% and 3.62%, respectively. Only 3.75% of civilized villages are distributed in areas above an altitude of 2000m. In terms of slope distribution, 98.45% of civilized villages in China are situated in regions with slopes of less than 20°. The largest number of villages are located below 5°, accounting for 79.44%. The percentage of civilized villages situated in regions with a slope of 5~10° is 11.36%, and the percentage of villages located in areas with a slope of 10~15° and 15~20° is relatively small, accounting for 5.53% and 2.12%, respectively. Only 1.55% of civilized villages are distributed in areas with slopes above 20°. Topography and geomorphology are the most crucial factors affecting the development of civilized villages, and different topographies and geomorphologies affect the population size, form, industry, and civilization construction in villages. In the plains area, the terrain is flat, the natural environment is good, the productivity is developed, the history of development is long, village settlements appear early and on a large scale, transportation and public service infrastructure construction is better, the socio-economic environment is more open, the external connection is strong, the development level of the village is higher, and the construction of rural civilization is optimum. However, the terrain in the plateau areas is rugged, the natural environment is harsh, the infrastructure is relatively poor, and the distribution of villages is scattered, which is not conducive to the construction of material and spiritual civilization in villages.
(2) Climatic conditions. Using the spatial coupling of civilized villages with the annual average temperature and precipitation in China, we found that they were distributed under different climatic conditions (Table 5). What’s more, the differences were obvious and they were mostly distributed in areas with mild temperatures and abundant precipitation. In terms of annual average temperature distribution, civilized villages are located in the areas of less than 5°C and 5~10°C, accounting for 10.12% and 14.14% respectively. The areas of 10~15°C and 15~20°C have a larger number of distributions, accounting for 35.61% and 32.61% respectively. In addition, 7.52% of civilized villages are distributed in areas where the annual average temperature is above 20°C. In terms of annual average precipitation distribution, civilized villages are distributed in the areas of less than 500mm, accounting for 14.37%. In the areas of 500~1000mm and 1000~1500mm, they account for 38.06% and 32.59% respectively, and in the area of 1500~2000mm, the percentage is 14.23%. In addition, only 0.75% of civilized villages are located in areas more than 2000mm. Regional natural climatic conditions are closely related to village construction, and temperature and precipitation are the main indicators reflecting regional climatic characteristics. Suitable temperature and precipitation not only provide sufficient light, heat, water and other energy and materials for the growth and development of crops in agricultural production, but also are necessary elements for the normal life of rural residents. Temperature and precipitation have a significant influence on the working efficiency and living comfort of rural residents, and suitable climatic conditions are more conducive to the formation of a good culture, which will help to boost the construction of rural civilization.
(3) Economic level. According to the GDP statistics of civilized villages in China, the result is shown in Fig 6. It can be seen that civilized villages are centrally distributed in areas with better economic development, while their distribution is sparse and fewer in areas with low GDP. In areas with a GDP of less than 2 million yuan/km^2^, the number of civilized villages is the lowest, accounting for about 10.14% of the total. In the regions of 2~5 and 5~10 million yuan/km^2^, the numbers are relatively stable, accounting for 13.18% and 13.66% respectively. Between 10 and 20 million yuan/km^2^, the number is higher, accounting for 17.60%; and in the region of more than 20 million yuan/km^2^, the number is the highest, accounting for about 45.42%. The level of regional economic development supports the construction of rural civilization. In economically developed regions, the mobility of labor and economic factors is high, the openness of cultural and social organizations is strong, the connection with neighboring regions is close and intertwined, the production and lifestyle are more advanced and modern, and the living standard and quality are higher. The residents no longer live materially, but have enough economic basis to lead a good spiritual and cultural life, which contributes to the construction of rural material civilization and spiritual civilization. On the contrary, regions with underdeveloped economies and low degree of openness of the territorial system, backward modes of production and difficult living conditions. In their development, they pay more attention to improving economic strength and neglect the improvement of ideological and cultural construction and social civilization.
(4) Transport location. Statistical analysis of the distance between civilized villages and national roads, provincial roads and county roads in China indicates that civilized villages are concentrated in areas closer to county roads and provincial roads (Table 6). The distribution pattern of the distance from national roads is not significant, and a larger proportion of civilized villages are distributed in areas far from national roads. The proportion of civilized villages within ≤5km, 5~10km, 10~15km, 15~20km and >20km of county roads is 59.41%, 17.70%, 8.27%, 5.20% and 9.42% respectively. The proportion of civilized villages within ≤5km, 5~10km, 10~15km, 15~20km and >20km of provincial roads is 57.46%, 19.55%, 10.09%, 4.77% and 8.13% respectively. The proportion of civilized villages within ≤5km, 5~10km, 10~15km, 15~20km and >20km of national roads is 32.61%, 15.99%, 11.53%, 9.02% and 30.85% respectively. The development and construction of civilized villages are dominated by human activities. Transport is the artery of economic growth and an essential element in determining the external links of a region, affecting the flow of people, materials and information in villages. In the process of village development, the transport location serves as a bridge for the external development and internal progress of civilized villages, and a crucial guarantee for the advancement of material and spiritual civilization. Areas with superior transport location, rich social resources and high feasibility of construction are more conducive to the progress of rural civilization.
(5) Policy-driven. Statistical analysis of the distance between civilized villages in China and county (town) centers shows that civilized villages are centrally distributed in areas closer to township centers (Fig 7a), but there is no obvious law between the distribution and county administrative centers (Fig 7b). Within 5km of the township center, the number of civilized villages is the highest, accounting for 68.74%. Within 5~10km, the amount is relatively large, accounting for 25.27%. The amounts in the ranges of 10~15km, 15~20km and more than 20km are particularly low, accounting for 3.32%, 1.12% and 1.55% respectively. Within 5km from the county administrative center, the number of civilized villages is the least, accounting for 12.91%. Within 5~10km and 10~15km, the quantity is relatively high, accounting for 22.41% and 22.04% respectively. Within 15~20km, it accounts for 16.73%; and beyond 20km, it is the highest, accounting for 25.91%. Driven by policies, human production and lifestyles constantly influence the development of villages, and the distance from towns can better reflect the impact of policies on rural civilization. In general, the shorter the distance between villages and towns, the more developed the communication is, the wider the channels of information, and the more vulnerable to political influence. Whether industry or society, the more open it is, the easier it will be to build and develop rural civilization. On the contrary, villages with poor political location conditions have fewer channels to obtain information and are weakly influenced by politics, which is not conducive to the construction of rural civilization as a whole.

**Table 4:** Distribution of civilized villages at different topography and geomorphology in China.

Topography and geomorphology	Type	The Number of Civilized Villages	Proportion
Elevation	≤500 m	3397	70.42%
	500~1000 m	598	12.40%
	1000~1500 m	473	9.81%
	1500~2000 m	175	3.62%
	>2000 m	181	3.75%
Slope	≤5°	3832	79.44%
	5~10°	548	11.36%
	10~15°	267	5.53%
	15~20°	102	2.12%
	>20°	75	1.55%

**Table 5:** Distribution of civilized villages at different climatic conditions in China.

Climatic conditions	Type	The Number of Civilized Villages	Proportion
Annual Average Temperature	≤5°	488	10.12%
	5~10°	682	14.14%
	10~15°	1718	35.61%
	15~20°	1573	32.61%
	>20°	363	7.52%
Annual Average Precipitation	≤500 mm	693	14.37%
	500~1000 mm	1836	38.06%
	1000~1500 mm	1572	32.59%
	1500~2000 mm	687	14.23%
	>2000 mm	36	0.75%

**Table 6:** Distribution of civilized villages at different roads in China.

Distance	County road		Provincial road		National road	
	The Number of Civilized Villages	Proportion	The Number of Civilized Villages	Proportion	The Number of Civilized Villages	Proportion
≤5 km	2866	59.41%	2772	57.46%	1573	32.61%
5~10 km	854	17.70%	943	19.55%	772	15.99%
10~15 km	399	8.27%	487	10.09%	556	11.53%
15~20 km	251	5.20%	230	4.77%	435	9.02%
>20 km	454	9.42%	392	8.13%	1488	30.85%

Overall, the distribution of civilized villages has perceptible regional differentiation laws in China, and the distribution features differ in distinct geographic contexts. Due to the flat terrain, fertile soil, suitable climatic conditions, relatively rich hydrothermal resources, optimum transportation infrastructure, and developed economy, the agglomeration and distribution characteristics of civilized villages are evident in the eastern region of the “Hu Huyong Line”. For example, the unique topographic conditions of the Sichuan Basin and its surrounding areas have created a warm and humid climate as well as fertile and productive soil, which provide a good living environment for the distribution of civilized villages, and a high-density agglomeration of civilized villages has been formed in this area. The Yangtze River Delta region is flat and open, with a dense river network and gentle water flow, making it suitable for farming. The high land productivity in this region has made it a significant agricultural production area in China. At the same time, the region has optimum transportation infrastructure, high internal and external connections, and a high degree of economic development; therefore, it has also formed a high-density agglomeration area of civilized villages. In contrast, the area west of the “Hu Huanyong Line” is characterized by poor natural resource conditions, poor living environments, and insufficient water and thermal conditions to satisfy the planting demands of most large-scale crops. The density of the transportation network is low, the level of economic development is backward, the state of village buildings is low, and the number of civilized villages is relatively small.

## 4. Discussion

Civilization of the countryside is both the focus and difficult part of the rural revitalization strategy. Since its reform and opening up, the country has focused on economic construction and urban development, and countryside civilization has not received enough attention for a long time, lacks corresponding investment, and has many problems. A civilized village is typically representative of a model village of rural civilization, which is a new model for the development of rural customs in the new era. Taking civilized villages as the research object, through the study of their spatiotemporal pattern evolution and geographic background, the spatiotemporal development law of the model villages of rural style and civilization, and the basic work of scientific cognition of the new characteristics of civilized village development in the era of rural revitalization can be discovered. The results of this study are of great theoretical significance and practical value for promoting the development of model villages for rural civilization, deepening the theory of the development of typical villages, and helping rural revitalization.

Compared to previous studies, this study focuses on civilized villages and analyzes their spatial distribution patterns in different periods with time as the axis. It was found that the spatial distribution of civilized villages was highly uneven, with 83.83% of civilized villages concentrated within an altitude range of 1000m, which is a high degree of agglomeration. The spatial analysis method was used to explore civilized villages from the macro perspective of geography, which provides a clearer understanding of the distribution patterns of civilized villages and enriches the research content of rural geography and rural revitalization.

The spatial and temporal distribution patterns of civilized villages are the result of comprehensive impact of multiple elements, including local geographic location, customs and habits, economic level, government policy, leadership, institutional culture, regional beliefs, and many other factors related to the development and distribution of civilized villages, as well as education levels, ideological and moral qualities, the human habitat environment, publicity and education, and infrastructure construction. Due to limitations in data availability and certain indicators, quantitative expression is difficult. This study only examines the influencing factors from the aspects of topography and geomorphology, climatic conditions, economic level, transport location, and policy drive and fails to fundamentally analyze the driving and internal mechanisms of the formation of civilized villages in terms of villages themselves. It is imperative to gather pertinent data for future studies to improve this research. Second, the efficiency and mechanism of civilized villages in promoting rural civilization and revitalization are worth further investigation. Future research should pay more attention to the impact of civilized villages on booming local industries, eco-livability, effective governance, and affluent life. It is also possible to choose diverse types of civilized villages as examples for empirical research and to discuss the main features and contents of civilized village construction to improve the pertinence measures for the development of rural civilization and thus achieve the effect of local conditions and benign development.

## 5. Conclusions

The civilization of rural areas is a basic requirement for the construction of a new countryside. It is a reflection of good governance in the countryside, an important task in the construction, and a powerful guarantee for the comprehensive revitalization of the countryside. Influenced by a combination of natural and human factors, the construction of rural civilization in China is still plagued by problems such as uncoordinated regional development and serious homogenization. As a product of the development of rural civilization, civilized villages are of considerable significance as a driving force for changing customs and establishing a new civilized style in rural China. The construction of civilized villages will also continue to change the traditional stereotypes and outdated customs in rural areas. This study takes the civilized villages as the topic and explores their spatial distribution characteristics in China and geographic context factors by using the nearest neighbor index, kernel density, as well as spatiotemporal rearrangement scanning statistical analysis. Compared with previous articles on villages, this study does not only research the distribution and evolution of villages from a temporal or spatial, but also employs the spatiotemporal scanning method to study the cold and hot spot areas of their spatiotemporal evolution, which is the innovation of this study. It is hoped that this study will provide a theoretical reference for enhancing the development of civilized villages and typical reference for deepening the study of rural revitalization in academia.

In terms of time evolution, the number of civilized villages has shown a significant upward trend, and the growth rate of each selection session is also increasing rapidly since 2005. In terms of spatial distribution, the spatial distribution pattern of civilized villages is more in the east and less in the west, more in the south and less in the north, which shows uneven distribution characteristics, and the difference between east and west is more significant than that between north and south. The center of gravity of the spatial distribution of civilized villages is always in Henan Province. The spatial distribution shows a significant agglomeration pattern, mainly forming some high-density areas dominated by Beijing-Tianjin-Hebei, the Yangtze River Delta, Sichuan-Chongqing and the border areas of Shanxi and Henan, presenting a multi-core spatial distribution pattern.

In terms of spatiotemporal agglomeration, civilized villages show clear spatiotemporal clustering characteristics. From 2005 to 2011, the growth rate of civilized villages in Ansai District, Zhidan County, Zichang City, and other areas was higher than that of the surrounding areas, forming a spatiotemporal hot spot cluster. From 2005 to 2008, the growth rate of civilized villages in Wuning County, Jing’an County, Tongshan County, and other areas was slower than that of the surrounding areas, forming a spatial and temporal cold spot cluster.

In terms of geographic contexts, civilized villages are geographically differentiated. Civilized villages are clustered in the eastern part of the country, where the terrain is flat, climatic conditions are favorable, water and heat resources are abundant, transport infrastructure is perfect and the level of economic development is high. In the western region, where conditions are weaker, there are fewer civilized villages.

## Supporting information

S1 Table Number statistics of civilized villages. (XLSX)
